# Laboratory readiness and response for novel coronavirus (2019-nCoV) in expert laboratories in 30 EU/EEA countries, January 2020

**DOI:** 10.2807/1560-7917.ES.2020.25.6.2000082

**Published:** 2020-02-13

**Authors:** Chantal B.E.M. Reusken, Eeva K. Broberg, Bart Haagmans, Adam Meijer, Victor M. Corman, Anna Papa, Remi Charrel, Christian Drosten, Marion Koopmans, Katrin Leitmeyer, on behalf of EVD-LabNet and ERLI-Net

**Affiliations:** 1 Centre for Infectious Disease Control, National Institute for Public Health and the Environment, Bilthoven, the Netherlands; 2 Viroscience department, Erasmus MC, Rotterdam, the Netherlands; 3 European Centre for Disease Prevention and Control, Solna, Sweden; 4 Charité - Universitätsmedizin Berlin Institute of Virology, Berlin, Germany; 5 German Centre for Infection Research (DZIF), Berlin, Germany; 6 Department of Microbiology, Medical School, Aristotle University of Thessaloniki, Thessaloniki, Greece; 7 Unité des Virus Emergents (Aix-Marseille Univ-IRD 190-Inserm 1207-IHU Méditerranée Infection), Marseille, France; 8 The participating members of EVD-LabNet and ERLI-Net are acknowledged at the end of the article

**Keywords:** 2019-nCoV, coronavirus, emerging infections, laboratory, response, zoonoses

## Abstract

Timely detection of novel coronavirus (2019-nCoV) infection cases is crucial to interrupt the spread of this virus. We assessed the required expertise and capacity for molecular detection of 2019-nCoV in specialised laboratories in 30 European Union/European Economic Area (EU/EEA) countries. Thirty-eight laboratories in 24 EU/EEA countries had diagnostic tests available by 29 January 2020. A coverage of all EU/EEA countries was expected by mid-February. Availability of primers/probes, positive controls and personnel were main implementation barriers.

In early January 2020, it became evident that a new pathogenic human coronavirus, provisionally named novel coronavirus (2019-nCoV), had emerged in China [1,2]. The virus is causing an outbreak, which started in the metropole Wuhan, but was seeded through travellers across China with ongoing secondary chains of transmission in a wider geographical area. As at 10 February 2020, 40,553 confirmed cases including 910 deaths have been reported worldwide with an increasing number of cases being reported in Europe [3]. So far, instances of secondary spread from international travellers have been limited, but clusters of human-to-human transmission have been reported involving persons with close contact to confirmed cases [4]. A key knowledge gap is the efficiency of community transmission of 2019-nCoV, including the contribution of mild or asymptomatic cases. On 30 January 2020, the Word Health Organization (WHO) declared the outbreak a public health emergency of international concern (PHEIC) because of these uncertainties, the ongoing seeding of the virus internationally, and the need for preparedness across the world in order to track and control the epidemic. WHO highlighted the crucial role of early detection of cases to interrupt virus spread and emphasised that countries need to put in place strong measures to detect and laboratory-confirm cases early [5]. Here, we assessed the required expertise and diagnostic capacity in specialised laboratories in 30 European Union/European Economic Area (EU/EEA) countries.

## Survey

**Figure 1:**
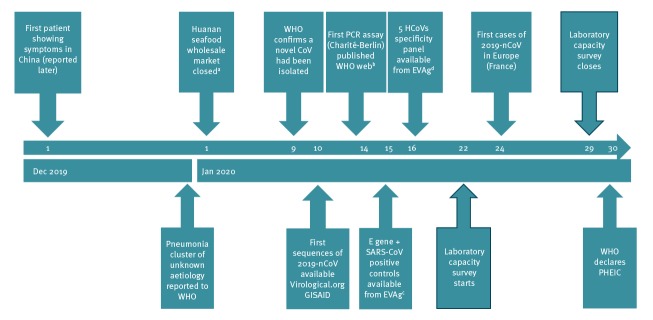
Time-line with hallmark events of the first two months of the novel coronavirus (2019-nCoV) outbreak, December 2019–January 2020

CoV: corona virus; E gene: envelope gene of 2019-nCoV; EVAg: European Virus Archive – GLOBAL; GISAID: Global Initiative on Sharing All Influenza Data; HCoVs: human coronaviruses; 2019-nCoV: novel coronavirus; PHEIC: public health emergency of international concern; SARS-CoV: severe acute respiratory syndrome corona virus; WHO: World Health Organization.

^a^ Of the initial 41 people who were hospitalised in Wuhan by 2 January 2020 with pneumonia due to a confirmed 2019-nCoV infection, 27 had an epidemiological link to the Huanan market [18].

^b^ https://www.who.int/docs/default-source/coronaviruse/protocol-v2-1.pdf?sfvrsn=a9ef618c_2

^c^ https://www.european-virus-archive.com/nucleic-acid/wuhan-coronavirus-2019-e-gene-control and https://www.europeanvirus-archive.com/nucleic-acid/sars-cov-frankfurt-1

^d^ The five HCoVs included HCoV-NL63, HCoV-OC43, HCoV-229E, MERS-CoV, SARS-CoV. More information is available at: https://www.european-virus-archive.com/nucleic-acid/coronavirus-rna-specificity-panel

A questionnaire was designed to assess the capacity, quality and operational specifics related to 2019-nCoV diagnostics, as well as barriers against their implementation in laboratories that are part of the European Centre for Disease Control and Prevention (ECDC)-associated European expert laboratory network for emerging viral diseases (EVD-LabNet) and/or the European Reference Laboratory Network for Human Influenza (ERLI-Net). The survey was sent on 22 January 2020 to the Operational Contact Points representing 81 laboratories in, among others, 30 EU/EEA countries. The survey subsequently closed on 29 January 2020 (Figure 1). Where indicated, data were validated by individual email exchange with the laboratories to include one entry per laboratory. Entries from laboratories outside the EU/EEA and veterinary laboratories were omitted from analysis for this report. In total, the data provided by 47 laboratories in 30 EU/EEA countries were taken into account in this study. 

## Capacity for novel coronavirus molecular diagnostics

**Figure 2:**
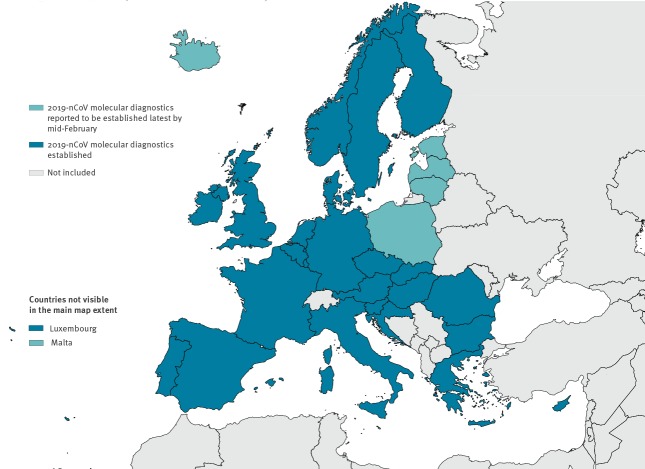
Status of availability of molecular diagnostics for novel coronavirus (2019-nCoV) in EU/EEA countries as at 29 January 2020 (n = 46 laboratories)^a^

At country level, 24 of 30 EU/EEA countries had already implemented molecular tests for 2019-nCoV while the laboratories in the remaining six countries had arranged to ship clinical specimens of suspected cases to a specialised laboratory abroad, while planning to implement assays between 30 January and 17 February 2020. At the laboratory level, 38 of 47 responding laboratories had implemented molecular diagnostics for 2019-nCoV at survey submission, and eight of the nine remaining laboratories planned to have tests implemented by mid-February 2020 (Figure 2). Nineteen laboratories indicated to have capacity to perform whole genome sequencing on 2019-nCoV in clinical samples, while 15 laboratories could perform partial sequencing.

EU/EEA: European Union/European Economic Area; 2019-nCoV: 2019 novel coronavirus.

^a^ One laboratory of the 47 included in the current study did not indicate when its molecular diagnostics would be available.

**Figure 3:**
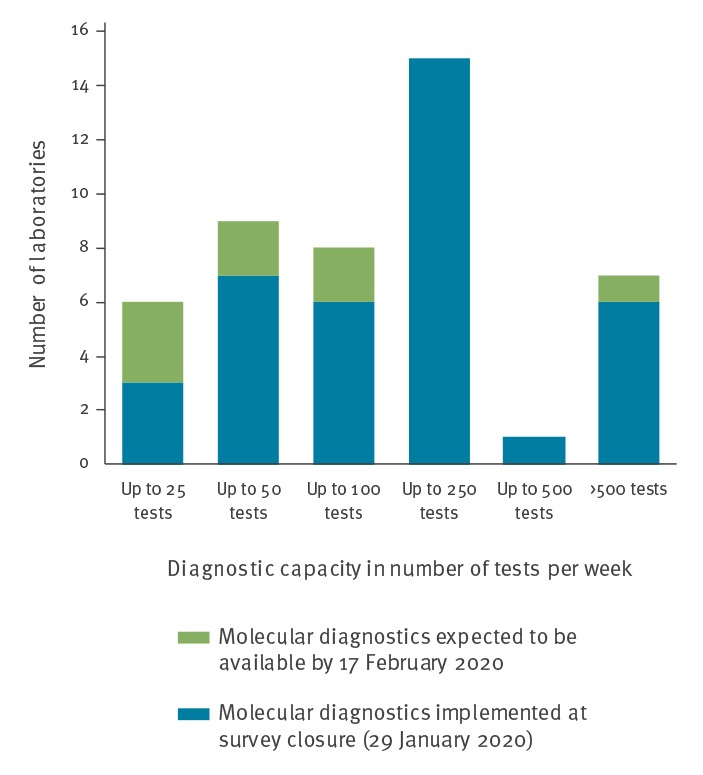
Diagnostic capacity of specialised laboratories with molecular tests available or forthcoming for novel coronavirus (2019-nCoV), EU/EEA, January 2020 (n = 46)^a^

The laboratories were asked to indicate their weekly capacity for molecular testing for 2019-nCoV (Figure 3). Overall, for all 38 laboratories with current capacity this was indicated to be at a minimum of 8,275 tests per week. The eight laboratories in the process of implementing molecular diagnostics would, all combined, add a minimum capacity of 875 tests per week once this process would be complete.

EU/EEA: European Union/European Economic Area.

^a^ One laboratory of the 47 included in the current study did not indicate when its molecular diagnostics would be available.

## Expertise for coronavirus and other respiratory pathogens

Forty-five laboratories in 28 countries indicated having previous expertise in human coronavirus (HCoV) diagnostics. For two countries the two responding laboratories had no experience. Twenty-five laboratories in 19 countries indicated having experience in molecular diagnostics for all six additional HCoVs (HCoV-HKU1, HCoV-OC43, HCoV-NL63, HCoV-229E, Middle East respiratory syndrome CoV and severe acute respiratory syndrome CoV) [6]. Forty-four laboratories in 29 countries performed differential testing for other common respiratory pathogens of viral and bacterial origin. Overall, the 47 survey respondents indicated their ability to process a wide range of respiratory sample types, including nasopharyngeal swabs (n = 38), bronchoalveolar lavage (n = 36), oropharyngeal swab (n = 34), nasopharyngeal aspirate (n = 34), sputum (n = 34), (endo) tracheal aspirate (n = 32) and nasal wash (n = 29). In addition, a number of respondents indicated that their laboratories could process biopsy materials (n = 28) and whole blood, plasma, serum (n = 28) for 2019-nCoV detection.

## Implementation of molecular diagnostics for novel coronavirus 

### Biosafety level

For the biosafety-level (BSL) applied for inactivation of clinical samples suspected of 2019-nCoV, 22 laboratories of the 47 EU/EEA laboratories indicated to do this at BSL2. Twenty-one laboratories indicated to do so at BSL3. Four laboratories indicated an intermediate level BSL2 + (BSL2 with extra precautions such as wearing a filtering face piece (FFP)2 mask). Different approaches were observed between laboratories within some countries.

### Test specifics

As of 14 January 2020, protocols for RT-PCR of 2019-nCoV are being published on the WHO website [7]. At survey closure (29 January 2020), the envelope (E)-gene screening test as published by Corman et al. [6,7], had been implemented by 35 laboratories and the confirmatory RNA-dependent RNA polymerase (RdRp)-gene test and nucleoprotein (N)-gene test by respectively 33 and 21 laboratories. Sixteen laboratories indicated to have additional tests, i.e. in house tests (n = 5), pan-CoV tests (n = 12) or an assay based on Poon et al. (n = 1) [7]. Two laboratories indicated to base 2019-nCoV testing solely on previously published pan-CoV tests [8]. 

### Level of specificity validation

Only 11 laboratories of the 38 laboratories that had implemented testing indicated having validated the specificity of the implemented test against the six additionally known HCoVs and other common respiratory pathogens. For 15 laboratories, specificity validation was still in progress at the time of data submission. Seven laboratories indicated to have only partially validated the implemented test(s) while five laboratories had not (yet) performed any validation. The questionnaire was send out before the first 2019-nCoV cases appeared in Europe (Figure 1) and positive clinical specimens were assumed to be not available to the European laboratories. Therefore, the level of validation for clinical sensitivity was not assessed.

### Positive control

Three of 38 laboratories that had implemented diagnostics did the implementation without a positive control. Indicated sources for positive controls were the European Virus Archive (EVAg) (synthetic 2019-nCoV E-gene, SARS-CoV RNA) (n = 23) [9], or own stocks, i.e. SARS-HCoV RNA and/or synthetic RNA (n = 15).

## Diagnostic challenges

**Figure 4:**
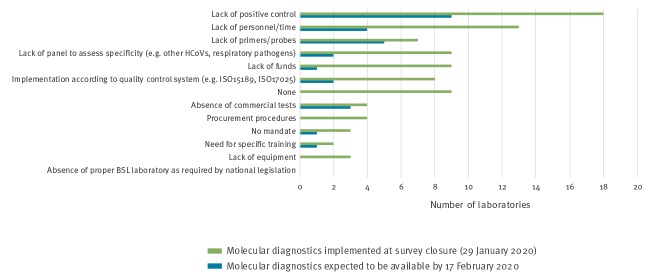
Challenges reported by laboratories in terms of implementing molecular diagnostics for novel coronavirus (2019-nCoV), EU/EEA, January 2020 (n = 47)

BSL: biosafety-level; EU/EEA: European Union/European Economic Area; HCoVs: human coronaviruses; ISO: International Organization for Standardization.

The top three challenges that were experienced for test implementation were an initial lack of positive control, lack of personnel/time and a lack of primers and/or probes (Figure 4). Nine laboratories in eight countries indicated no obstacles.

## Discussion

As at 10 February 2020, 37 confirmed 2019-nCoV cases were reported from eight European countries based on ECDC reporting and testing criteria [3,10]. Multiple modelling studies estimated the risk of 2019-nCoV introduction to Europe as high [11-14]. Pullano et al. indicated the United Kingdom, France and Germany as being at the highest risk, followed by Italy, Spain and the Netherlands [11]. Indeed, all but one country (the Netherlands) have reported cases. The study reported that the occurrence of 2019-nCoV importation from Beijing and Shanghai, both cities with high numbers of travellers to Europe, would likely lead to an even higher and widespread risk for Europe.

This rapid assessment of the readiness of EU/EEA laboratories for molecular detection of 2019-nCoV demonstrated a fast implementation of molecular diagnostics by the European specialised laboratory networks with a good geographical coverage for testing. Among both laboratory networks in this study, protocols were shared rapidly and there was an early availability of positive controls and CoV specificity panels via EVAg. Furthermore, the survey indicated a great willingness of laboratories to provide international diagnostic support [10] and to share sequences to contribute to the monitoring of virus evolution and trace transmission chains.

However, although the first protocols suggesting primer/probe sequences were available fast through the WHO website (Figure 1) and validation panels were made available through the EVAg portal soon after [6,7], the availability of primers, probes and positive controls were indicated as most important initial obstacles for test implementation. In addition, lack of sufficient personnel to implement and validate was a barrier, as had been observed in response to the Zika virus (ZIKV) outbreak in the Americas and the related PHEIC [15]. This suggests that the challenges faced by specialised laboratories when responding to emerging events are of structural nature.

Capacity-wise, the survey indicates that European specialised laboratories are prepared for the current situation, and suggests that a more sensitive case definition than currently in use [10,16] would not create an immediate bottleneck. However, it remains to be seen how realistic the estimates are, particularly in view of the coinciding seasonal epidemic peaks of other respiratory pathogens such as influenza viruses. This will depend on the epidemiological developments in the 2019-nCoV outbreak and on whether the current worldwide control strategy of containment with active case finding [5] will be sustained and the indicated laboratory capacity will suffice. If the outbreak turns into a pandemic, specialised laboratories’ efforts would refocus to reference activities like confirmatory testing, laboratory surveillance including virus characterisation, provision of reference materials and advice, while general testing for 2019-nCoV would shift to first-line hospital laboratories that currently do not have this capacity. This would require a roll-out of tests from the specialised laboratories as was done during the 2009 influenza A(H1N1) pandemic.

The survey showed that proper validation of specificity was lacking in a vast majority of the laboratories that had implemented testing while very few laboratories indicated to have implemented tests without availability of a positive control. The important assessment of the clinical sensitivity of the implemented tests was not possible in this very early phase of laboratory response due to the, at the time, absence of positive clinical materials in Europe. The three laboratories without a positive control will also not have assessed the analytical sensitivity of their tests. The legal possibilities (General Data Protection Regulation; GDPR) for sharing and the willingness to share positive clinical material among the network laboratories now that the first 37 cases have been confirmed in the EU/EEA will determine the speed with which laboratories can address the clinical sensitivity of their implemented tests while the number of cases in the EU/EEA is still limited.

To properly assess the actual capability of the laboratories to detect (sub)clinical 2019-nCoV cases and to provide directions for corrective actions, proficiency testing by external quality assessment (EQA) is essential and urgently needed. The importance of EQA was illustrated in the European ZIKV response where timely implementation was not matched by an overall good capability [17]. Forty of the 47 responding laboratories in this study indicated that they will participate in such an assessment. Currently activities are ongoing to assess the actual capabilities within both laboratory networks by EQA.

In conclusion, while molecular testing for 2019-nCoV was quickly implemented in EU/EEA countries there is still room for improvement especially in the aspect of clinical validation of specificity and sensitivity, as could be expected considering the survey was taken in the very early phase of the laboratory response. Capability testing based on proficiency panels is needed.

## Editorial note

This article was published as an e-Alert on 11 February 2020.
